# Multiplexed SERS Detection of Serum Cardiac Markers Using Plasmonic Metasurfaces

**DOI:** 10.1002/advs.202405910

**Published:** 2024-10-15

**Authors:** Peng Zheng, Lintong Wu, Piyush Raj, Jeong Hee Kim, Santosh Kumar Paidi, Steve Semancik, Ishan Barman

**Affiliations:** ^1^ Department of Mechanical Engineering Johns Hopkins University Baltimore MD 21218 USA; ^2^ Biomolecular Measurement Division Material Measurement Laboratory National Institute of Standards and Technology Gaithersburg MD 20899 USA; ^3^ Department of Oncology Johns Hopkins University School of Medicine Baltimore MD 21287 USA; ^4^ The Russell H. Morgan Department of Radiology and Radiological Science Johns Hopkins University School of Medicine Baltimore MD 21287 USA

**Keywords:** cardiac biomarkers, multiplexed detection, plasmonic metasurface, Raman spectroscopy, SERS

## Abstract

Surface‐enhanced Raman spectroscopy (SERS) possesses exquisite molecular‐specific properties with single‐molecule sensitivity. Yet, translation of SERS into a quantitative analysis technique remains elusive owing to considerable fluctuation of the SERS intensity, which can be ascribed to the SERS uncertainty principle, a tradeoff between “reproducibility” and “enhancement”. To provide a potential solution, herein, an integrated multiplexed SERS biosensing strategy is proposed, which features two distinct advantages. First, a subwavelength‐structured plasmonic metasurface consisting of alternately stacked metal–dielectric pyramidal meta‐atoms is fabricated and could provide simultaneously enhanced electric and magnetic fields to enable spatially extended and weakly wavelength‐dependent SERS. Second, nanomechanical perturbations are harnessed to transduce signals in the form of SERS frequency shifts, which are not directly affected by the SERS uncertainty principle. By also employing 3D printing methods, a proof‐of‐concept study of multiplexed detection of a panel of serum cardiac biomarkers for acute myocardial infarction is provided. Success in the development of both the electric and magnetic fields‐active plasmonic metasurfaces could transform future designs of SERS substrates with newly endowed functionalities, and frequency shift‐based SERS multiplexing could open new opportunities to develop innovative quantitative optical techniques for applications in chemistry, biology, and medicine.

## Graphical abstract

This study presented an innovative SERS biosensing strategy which combines subwavelength‐structured plasmonic metasurfaces and the use of SERS frequency shifts for multiplexed detection of a panel of serum cardiac biomarkers for acute myocardial infarction, with practically useful and clinically relevant performance.

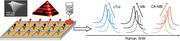

## 1 Introduction

As a noninvasive and noncontact analytical technique with distinct molecular fingerprinting capability and single‐molecule sensitivity, surface‐enhanced Raman spectroscopy, or SERS, has recently dramatically advanced biomedical diagnostics and therapeutics,^[^ ^1^, ^2^, ^3^ ^]^ accelerated the development of biopharmaceuticals,^[^ ^4^, ^5^ ^]^ facilitated detection of antimicrobial resistance,^[^ ^6^, ^7^ ^]^ and found promising applications in areas including forensic science^[^ ^8^ ^]^ and homeland security.^[^ ^9^ ^]^ Despite its great promise, the translation of SERS into a quantitative analysis technique in clinical settings^[^ ^10^ ^]^ is hindered by two major challenges. First, despite the electromagnetic (EM) field enhancement mechanism, the prevailing SERS techniques primarily rely on the electric field (E‐field) component to amplify the intrinsically inefficient Raman scattering by accelerating the vibrational transition dynamics, while the magnetic field (H‐field) component remains largely unexplored.^[^ ^1^, ^11^ ^]^ The absence of the H‐field component in SERS enhancement can be attributed to the weak permeability of most metals at optical frequencies, and this asymmetric contribution of the EM field to SERS highlights the incompleteness of the widely accepted EM enhancement mechanism, and underscores the untapped potential of the largely ignored H‐field in producing enhancement mechanism. Second, despite single‐molecule sensitivity, quantitative SERS analysis is constrained by the SERS intensity uncertainty principle, which is manifested as a considerable fluctuation of SERS signals that are amplified by intense hotspots.^[^ ^12^, ^13^, ^14^ ^]^ There are two primary mechanisms underlying the SERS intensity fluctuations. One is the spatial heterogeneity of SERS hotspots, which are mostly located at sparsely distributed sharp vertices and edges and narrow gaps with subnanometer dimensions.^[^ ^15^, ^16^, ^17^, ^18^, ^19^ ^]^ Such intense SERS hotspots cover far less area than do the bonded Raman molecules on plasmonic substrates. Consequently, only a small fraction of Raman molecules present contributes to most of the detected SERS signals. The other mechanism relates to complex dynamic processes involving the interplay between Raman molecules and the plasmonic substrate, which include but are not limited to the molecular adsorption, detachment, diffusion, and reorientation, as well as the possible atomic reconstruction of the SERS hotspots.^[^ ^12^, ^13^, ^14^ ^]^ As the distribution of the H‐field is spatially complementary to that of the E‐field, given the origin of the E‐field from surface polarization charges and the H‐field origin from fictitious current loops enclosed by those surface polarization charges, simultaneously enhanced E‐ and H‐fields could potentially alleviate the SERS intensity uncertainty possibly by providing denser SERS hotspots. One way to approach this goal is the development of plasmonic substrates containing sites that are both E‐ and H‐fields active.

Recently, photonic metamaterials have emerged as a promising platform for manipulating EM fields over a wide frequency range.^[^ ^20^, ^21^, ^22^ ^]^ Photonic metamaterials are artificially engineered media consisting of nanoscale constituent meta‐atoms patterned as subwavelength‐structured arrays and can exhibit novel optical effects not commonly observed in nature.^[^ ^20^ ^]^ Particularly, as opposed to most naturally occurring materials which only support significant magnetism up to a certain frequency in the gigahertz range,^[^ ^23^ ^]^ photonic metamaterials can be made to exhibit considerable optical magnetism by rationally structuring the meta‐atoms, and therefore they hold great promise to provide a highly desirable H‐field, complementary to the E‐field, for alleviating the spatial heterogeneity of SERS hotspots. Historically, split ring resonators have underscored the initial realization of such effects and catalyzed the development of artificial magnetism from the microwave to optical frequencies.^[^ ^23^, ^24^ ^]^ Recently, layered hybrid metal–dielectric nanoconstructs were established as favorable meta‐atoms for creating more SERS‐active plasmonic metasurfaces.^[^ ^25^, ^26^, ^27^ ^]^ They simultaneously support significant and spatially complementary E‐ and H‐fields, afford fine tuning of the EM properties, and allow manufacturing with ease and at scale. Importantly, the simultaneous excitation of spatially complementary E‐ and H‐fields yields SERS hotspots of denser population on the plasmonic metasurface, thus mitigating the spatial heterogeneity of SERS hotspots, and improving the consistency of the SERS intensity‐based signals. Nevertheless, intensity‐based SERS signals are intrinsically limited by molecular blinking and dynamic behaviors of molecule‐metal binding, highlighting the long‐standing challenge in translating SERS into a quantitative spectroscopic tool.^[^ ^12^, ^28^, ^29^ ^]^

While SERS is subject to intensity variation, its frequencies have been recently recognized as robust signal outputs that are less susceptible to fluctuation.^[^ ^30^, ^31^, ^32^, ^33^, ^34^, ^35^, ^36^ ^]^ This is because the frequencies of the SERS peaks correspond to the energy of a particular vibrational transition, and are not directly related to the magnitude of the SERS intensity or the number of molecules in hotspots. Previous research has established that nanomechanical perturbations induced by antibody‐antigen interactions can lead to structural deformation of the antibody‐conjugated Raman molecules, which results in a characteristic frequency shift in SERS.^[^ ^30^ ^]^ Notably, the frequency‐based SERS immunoassays only require the capture antibody for specific detection of antigen analytes. As compared to the prevailing sandwich immunoassays that require both capture and detection antibodies, the use of a single type of antibody significantly simplifies the immunoassay design. This can further eliminate the repeated incubation and washing steps that are often time‐consuming and demand additional hardware, and can thereby potentially lower the cost to shrink the gap of health inequity. Such a unique SERS frequency shift‐based method that requires only a single type of antibody has been utilized to create novel bioanalytical tools for detecting a wide range of biological analytes, such as small and macro‐molecules,^[^ ^37^, ^38^, ^39^ ^]^ proteins,^[^ ^31^, ^32^, ^33^ ^]^ and even circulating tumor DNA^[^ ^34^ ^]^ and serum microRNA^[^ ^35^, ^36^ ^]^ where the detection antibodies were replaced with single‐stranded DNA sequences that are complementary to those nucleic acid analytes. Therefore, the monitoring of SERS frequency shifts provides an alternative that holds great promise for achieving quantitative SERS analysis, unaffected by the uncertainty of SERS intensity measurements.

In this study, we combine SERS‐active plasmonic metasurfaces with the monitoring of SERS frequency shifts to develop a single‐antibody SERS biosensing platform for multiplexed detection of a panel of cardiac biomarkers in serum. Creatine kinase‐myocardial band (CK‐MB), myoglobin (Mb), and cardiac troponin‐I (cTnI) are three key biomarkers of acute myocardial infarction (AMI).^[^ ^40^, ^41^, ^42^ ^]^ Prompt and accurate diagnosis of AMI can inform immediate medical intervention, particularly reperfusion therapy, which can be crucial to improving patient outcomes.^[^ ^40^, ^43^ ^]^ While intensity‐based SERS approaches were previously explored for detecting cardiac biomarkers,^[^ ^42^, ^44^, ^45^, ^46^ ^]^ the potential of the integrated strategy combining frequency shifts in SERS with both the E‐ and H‐fields active plasmonic metasurfaces for multiplexed detection of serum cardiac biomarkers has not been reported. To fill this gap, first we design and fabricate a pyramidal plasmonic metasurface consisting of alternately stacked gold‐silica meta‐atoms. Finite‐difference time‐domain (FDTD) simulations were utilized to unambiguously demonstrate the spatially extended and weakly wavelength‐dependent SERS enhancement, which is contributed by both the E‐ and H‐fields. 3D printing was utilized to create a compartmentalized biosensing platform on the plasmonic metasurface. This provides an alternative approach to significantly increase the number of spots available for analysis on the same substrate, whereas regeneration strategies were traditionally utilized to address the infamous SERS memory effect.^[^ ^47^ ^]^ The SERS memory effect is an interference involving irreversible biochemical processes for measurements performed on the same substrate. Our new method enables simultaneous, high‐throughput, and cross interference‐free detection of multiple serum samples containing different cardiac biomarkers of varied concentrations.

## 2 Results and Discussion

### 2.1 Fabrication of Plasmonic Metasurfaces

**Figure 1:**
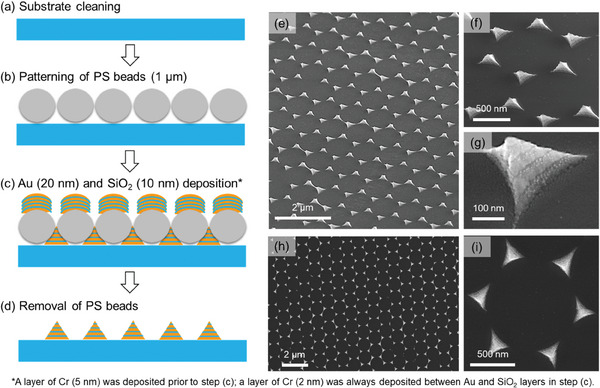
Fabrication and characterization of the gold–silica–gold alternately layered pyramidal plasmonic metasurfaces. a–d) Experimental protocol for fabricating the plasmonic metasurface on quartz substrates. e–g) Tilted‐view and h,i) top‐view SEM images of the fabricated plasmonic metasurfaces. The gold–silica‐layered structures can be seen in (f), (g), and (i) micrographs.

The plasmonic metasurfaces were fabricated based on nanosphere lithography, as schematically shown in **Figure** **1**, which has been well established in our previous studies.^[^ ^37^, ^38^, ^39^ ^]^ Detailed fabrication protocol can be found in Section S2 (Supporting Information). Briefly, following the quartz substrate cleaning and patterning of monolayer polystyrene beads (1 µm in diameter), gold and silica thin layers (20 and 10 nm in thickness, respectively) were alternately deposited by e‐beam evaporator to fill the gaps defined by the hexagonally patterned polystyrene beads. A total of five thin‐film layers of gold and four layers of silica were alternately deposited. Because of the poor adhesion between the metal and dielectric, a thin layer of chromium with a nominal thickness of 5 nm was deposited as the initial step, and then an ultrathin layer of chromium with a nominal thickness of 2 nm was deposited between each subsequent gold and silica deposition (Figure 4c). Given such a small thickness, we note that these chromium layers are unlikely to be continuous. Instead, they are more likely to be discrete nanoparticles and perhaps could be fused with part of the gold and silica layers. Based on SEM characterizations, the fabricated plasmonic metasurfaces display a hexagonally periodic pattern (Figure 1e,f,h,i), with each constituent meta‐atom having a pyramidal shape with sharp vertices and edges (Figure 1g–i). While the pyramidal geometry is reminiscent of solid metallic nanopyramid arrays we have studied previously,^[^ ^37^, ^38^, ^39^ ^]^ the well‐defined metal–dielectric layers (Figure 1f,g,i) are characteristic of the plasmonic metasurfaces. Consequently, the capacitive plasmonic coupling at the metal–dielectric‐metal interlayer^[^ ^48^, ^49^ ^]^ can be expected to dramatically alter the spatial EM field profiles for amplifying signals in SERS.

### 2.2 Spatially Extended and Simultaneously Enhanced E‐ and H‐Fields for SERS Enhancement

**Figure 2:**
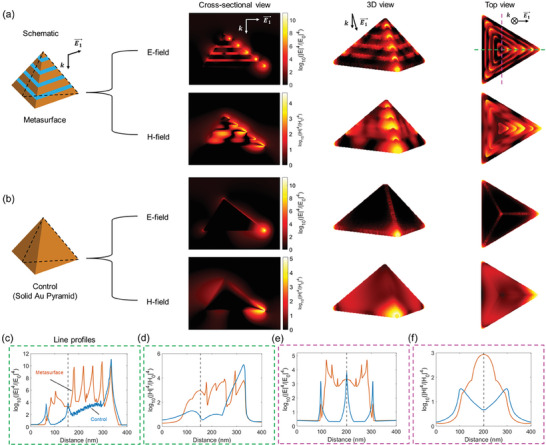
FDTD calculations of the E‐ and H‐fields for SERS enhancement on the plasmonic metasurface and Au nanostructure. a) From left to right: schematic of the plasmonic metasurface, cross‐sectional, 3D, and top view of the spatial profiles of SERS enhancement from the E‐field (upper panel) and H‐field (lower panel) components with an incident wavelength of 785 nm. b) The same as (a) except the metasurface is replaced by a solid Au pyramid with the same dimension as a control. c–f) Comparison of the line profiles of SERS enhancements between the plasmonic metasurface (red) and the control (blue) for both the E‐ and H‐fields extracted from the green and pink dashed lines in the top view. The SERS enhancement is calculated as $\frac{{\left|E\right|}^{4}}{{|E_{0}|}^{4}}$ for the E‐field component and $\frac{{\left|H\right|}^{4}}{{|H_{0}|}^{4}}$ for the H‐field component at the incident wavelength with the Stokes shift ignored and represented by $\mathrm{lo}g_{10}\frac{{\left|E\right|}^{4}}{{|E_{0}|}^{4}}$ or $\mathrm{lo}g_{10}\frac{{\left|H\right|}^{4}}{{|H_{0}|}^{4}}$ for easy spatial visualization (as shown in the colorbars).

To investigate how the EM field profiles were modified by the plasmonic metasurfaces as compared to previously studied solid gold nanopyramid arrays, FDTD numerical simulations were implemented to extract both the E‐ and H‐fields at an incident wavelength of 785 nm. In FDTD simulations, to be consistent with the nominal dimension of the fabricated plasmonic metasurfaces, an alternating gold‐silica layered nanopyramid with a base length of about 312 nm was studied while a solid gold nanopyramid with the same dimension was used as a control; these structures are shown schematically in **Figure** **2**a,b. All the EM field enhancement factors were converted to the SERS enhancement factors at the incident wavelength without considering the Stokes shift, and represented by the common logarithm for easy visualization, which is $\mathrm{lo}g_{10}\frac{{\left|E\right|}^{4}}{{|E_{0}|}^{4}}$ for the E‐field component and $\mathrm{lo}g_{10}\frac{{\left|H\right|}^{4}}{{|H_{0}|}^{4}}$ for the H‐field component. It was observed that, for both the E‐ and H‐fields, the plasmonic metasurface displayed spatially extended SERS enhancements, featuring a series of intense hotspots at the dielectric layers along both the edges and facets, which could be observed in the cross‐sectional, 3D, and top views in Figure 2a. In comparison, SERS enhancements were mostly concentrated at the vertex for the solid gold nanopyramid (Figure 2b). To quantify how the SERS enhancements differed spatially, we compared the line profiles of the SERS enhancements extracted along the dashed green and pink lines from the top views (Figure 2c–f). For the E‐field contribution to SERS, the plasmonic metasurface was found to display a series of SERS enhancement peaks originating from the capacitive plasmonic coupling at the gold‐silica‐gold interlayers, which were not seen on the solid gold nanopyramid (Figure 2c,e). For the H‐field contribution to SERS, the plasmonic metasurface exhibited similarly spatially extended and larger SERS enhancements as compared to the control. These observations highlight the unique spatially extended SERS enhancements supported by the plasmonic metasurface, not seen in conventional plasmonic nanostructures, such as the solid gold nanopyramid.

**Figure 3:**
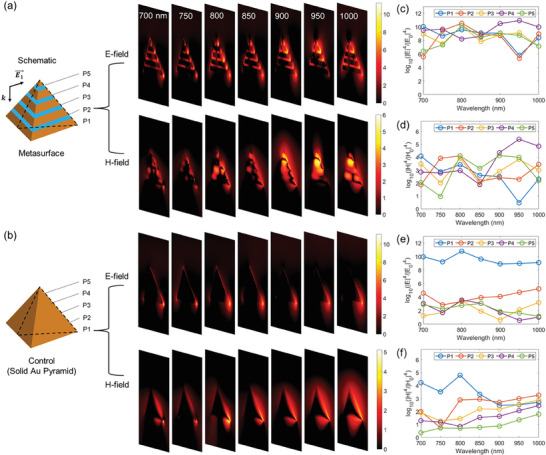
Wavelength‐dependent SERS enhancement. a) From left to right: schematic of the plasmonic metasurface, cross‐sectional view of the spatial profiles of SERS enhancement from the E‐field (upper panel) and H‐field (lower panel) components with varying incident wavelength. b) Similar representation as (a) except now for a solid Au pyramid with the same dimension, as a control. c–f) Comparison of the wavelength‐dependent SERS enhancement at different points (marked P1–P5 as shown in the schematic in (a) between the plasmonic metasurface (c and d) and in the schematic (b) for the control (e and f), for both the E‐ and H‐fields. The SERS enhancement is calculated as $\frac{{\left|E\right|}^{4}}{{|E_{0}|}^{4}}$ for the E‐field component and $\frac{{\left|H\right|}^{4}}{{|H_{0}|}^{4}}$ for the H‐field component at the incident wavelength with the Stokes shift ignored and represented by $\mathrm{lo}g_{10}\frac{{\left|E\right|}^{4}}{{|E_{0}|}^{4}}$ or $\mathrm{lo}g_{10}\frac{{\left|H\right|}^{4}}{{|H_{0}|}^{4}}$ for easy spatial visualization (as shown for the colorbars).

The wavelength‐dependent SERS enhancement was also studied to understand how the plasmonic metasurface compares with the solid gold nanopyramid over the wavelength range from 700 nm to 1000 nm. In particular, we focused on the variation of the SERS enhancement at the plasmonic hotspots marked as P1–P5, as schematically shown in **Figure** **3**a,b. While the SERS enhancement factors from the E‐field component were found to be weakly wavelength dependent for both the plasmonic metasurface and the solid gold nanopyramid (Figure 3c,e), the SERS enhancement factors from the H‐component were found to have a considerable fluctuation over the studied wavelength range (Figure 3d,f). But as different hotspots were found to have a different dependence on the wavelength, they could compensate each other and enable an overall high signal level and weak wavelength dependence of the SERS enhancement factors for the plasmonic metasurface. Such an ensemble contribution to SERS from all these hotspots combined is very different than the singular hotspot at the vertex of the solid gold nanopyramid. Collectively, the above studies demonstrated the unique capability of the plasmonic metasurface in enabling spatially extended and weakly wavelength‐dependent SERS enhancement.

### 2.3 Separate Detection of Each Type of Cardiac Biomarkers in Serum

**Figure 4:**
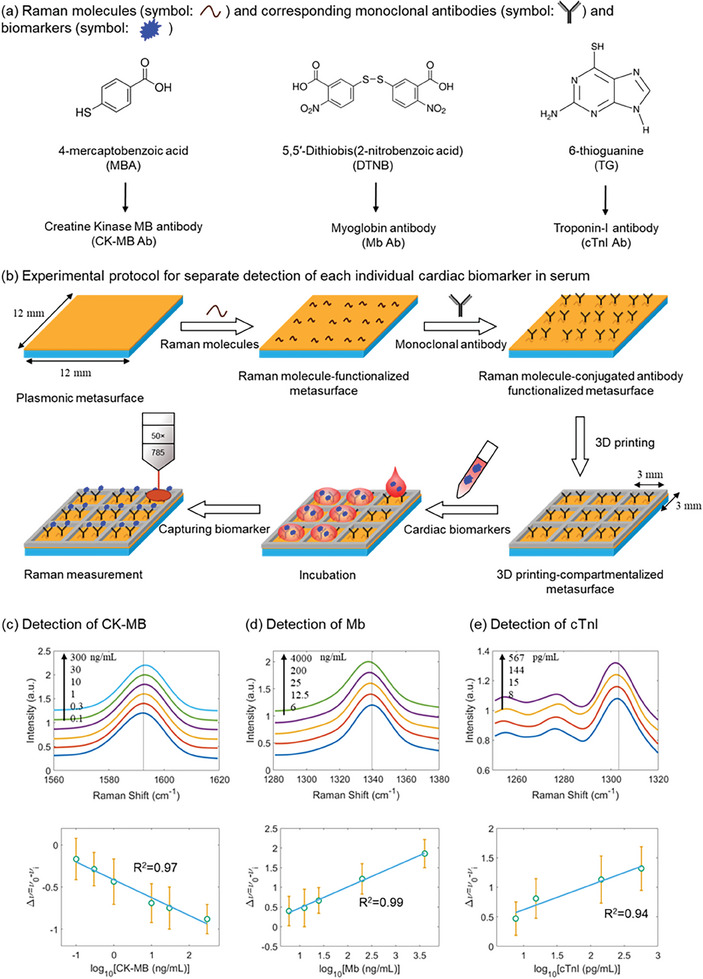
Separate detection of each type of cardiac biomarkers in serum. a) Raman molecules and corresponding monoclonal antibodies. b) Experimental protocol for separate detection of each type of cardiac biomarkers in serum. SERS spectra depicting the frequency shift of the peak of interest (top panel) and the regression analysis for the relative frequency shift with respect to the concentration of the detected cardiac biomarker (lower panel) for detection of c) CK‐MB, d) Mb, and e) cTnI. In (b), thermoplastic polyurethane was used as the 3D printing material. Error bars indicate the standard deviation of the mean frequency shifts for the studied concentration. The relative frequency shift Δν was defined as Δν  = ν_0_  − ν_*i*_, where ν_*i*_ is the frequency measured for a sample with a biomarker concentration of *i* and ν_0_ is that for the sample without any biomarkers.

After elucidating the SERS enhancement mechanism of the plasmonic metasurface, we proceeded to examine its performance as a biosensing platform for detecting a panel of serum cardiac biomarkers. Conventional SERS intensity‐based immunoassays are vulnerable to signal fluctuations.^[^ ^12^, ^13^, ^14^ ^]^ We adopted a frequency shift‐based spectro‐immunoassays design, which relies on a detection antibody to capture the antigen of interest and transduces a signal in the form of a frequency shift.^[^ ^30^, ^37^, ^38^, ^39^ ^]^ Three metasurface quartz substrates were used in our separate SERS detection studies. The three Raman molecules used for these sensing studies were 4‐mercaptobenzoic acid (MBA), 5,5′‐dithiobis‐(2‐nitrobenzoic acid) (DTNB), and 6‐thioguanine (TG), with each devoted to detecting a specific cardiac biomarker (CK‐MB, Mb, or cTnI, respectively) by including a conjugated antibody for target capture. The Raman molecules and associated capture antibodies are summarized in **Figure** **4**a, and the experimental protocol which we recently established is indicated in Figure 4b.^[^ ^37^, ^38^, ^39^ ^]^ Raman molecules were first functionalized on each of the three plasmonic metasurface substrates through Au–S covalent bonding. The corresponding monoclonal antibodies were then immobilized onto the plasmonic metasurfaces through the functionalized Raman molecule using carbodiimide crosslinker chemistry.^[^ ^50^ ^]^ Subsequently, 3D printing was implemented to compartmentalize each of the three plasmonic metasurfaces. Thermoplastic polyurethane (TPU) filament was used and heated to 228 °C at the extrusion nozzle for 3D printing. The printed compartments have a dimension of about 3 mm × 3 mm and are separated by a TPU wall with a thickness of about 1 mm or less. Each formed compartment could then be utilized to study a particular serum sample without cross interference. Serum samples containing different concentrations of one type of cardiac biomarker were pipetted into each compartment on the three differently functionalized plasmonic metasurfaces. After incubation at 37 °C for 20 min, excessive reagents were washed away using PBS buffer and the substrates were dried with compressed air. A total of 5 × 5 spectra were collected across an area of 20 µm × 20 µm in each compartment under an excitation wavelength of 785 nm. Each spectrum was collected with an acquisition time of 10 seconds and three accumulations.

The SERS spectra acquired for varying concentrations of CK‐MB, Mb, and cTnI were averaged and presented in the upper panel of Figure 4c–e, respectively. SERS spectra for Figure 4c–e with an extended wavenumber range are presented in Figure S1. The mean SERS spectra were vertically offset for better visualization. Herein, for detecting CK‐MB, we focused on the SERS peak of MBA at about 1590 cm^−1^ (Figure 4c), which originates from the C‐C breathing mode.^[^ ^30^ ^]^ For detecting Mb, we focused on the SERS peak of DTNB at about 1340 cm^−1^ (Figure 4d), which can be assigned to the N–O stretching mode.^[^ ^35^ ^]^ For detecting cTnI, we focused on the SERS peak of TG at about 1300 cm^−1^ (Figure 4e), which is from the ring C–N stretching mode.^[^ ^51^ ^]^ These characteristic SERS peaks, rather than the entire spectra, from the three Raman molecules were found to undergo biomarker‐induced frequency shifts owing to the nanomechanical perturbations,^[^ ^30^, ^32^, ^33^, ^34^, ^36^, ^51^ ^]^ and therefore, each of them was utilized to detect a particular type of serum cardiac biomarker. The relative frequency shift Δν was defined as Δν  = ν_0_  − ν_*i*_, where ν_*i*_ is the frequency measured for a sample with a biomarker concentration of *i* and ν_0_ is that for the sample without any biomarkers. Although the specific mechanism underlying frequency shifts in SERS has yet to be fully understood, we observed unequivocal frequency shifts correlated to the concentration variations for the antigen analytes, as shown in Figure 4c–e. The MBA molecules displayed a red shift at the characteristic SERS peak at about 1590 cm^−1^ with an increasing concentration of CK‐MB, which was consistent with previous studies of influenza‐H1 antigen and human p53 protein,^[^ ^30^ ^]^ protein carbonylation,^[^ ^32^ ^]^ and serum microRNA.^[^ ^35^, ^36^ ^]^ Both the DTNB and TG Raman molecules exhibited a blue shift at their respective characteristic SERS peaks at about 1340 and 1300 cm^−1^ with increasing concentrations of Mb and cTnI, consistent with recent studies of circulating tumor DNA^[^ ^34^ ^]^ and our studies of thyroid‐stimulating hormone.^[^ ^37^, ^38^, ^39^ ^]^ The correlation between frequency shifts in SERS and analytes’ concentrations was confirmed by the regression analysis, which returned coefficients of determination R^2^ of 0.97, 0.99, and 0.94 for the detection of CK‐MB, Mb, and cTnI, respectively. These coefficients of determination are overall better than those obtained on the solid gold nanopyramid for detecting thyroid‐stimulating hormone we studied previously,^[^ ^39^ ^]^ implying the advantage of the plasmonic metasurface‐based biosensing platform. The linear detection ranges for CK‐MB, Mb, and cTnI were found to be from 0.1 to 300 ng/mL, 6 to 4000 ng/mL, and 8 to 567 pg/mL, respectively. Based on the definition of the limit of detection (LOD) being 3σ/S, where σ is the standard deviation of blank samples, and S is the slope of the calibration curve, the LOD for separate detection of CK‐MB, Mb, and cTnI is about 0.04 ng/mL, 3.6 ng/mL, and 5.2 pg/mL, respectively. As a comparison, the normal concentrations for these three cardiac biomarkers are 0.3 to 4 ng/mL,^[^ ^52^ ^]^ 50 ng/mL or less,^[^ ^53^ ^]^ and 40 pg/mL or less.^[^ ^52^ ^]^ These results validate the potential for utilizing the frequency shifts in SERS on the plasmonic metasurface for separate detection of serum cardiac biomarkers.

### 2.4 Multiplexed Detection of Cardiac Biomarkers in Serum

**Figure 5:**
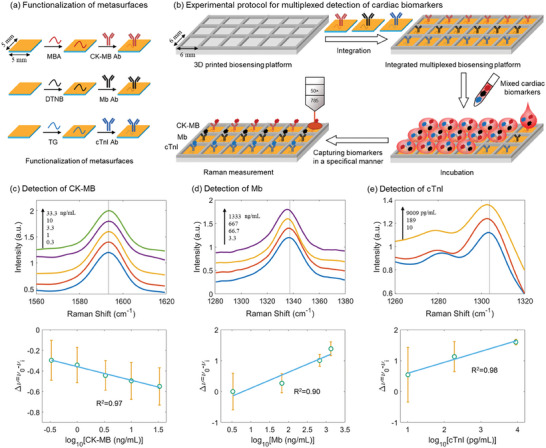
Multiplexed detection of cardiac biomarkers in serum. a) Functionalization of the plasmonic metasurfaces. b) Experimental protocol for multiplexed detection of cardiac biomarkers. SERS spectra depicting the frequency shift of the peak of interest (top panel) and the regression analysis for the relative frequency shift with respect to the concentration of the detected cardiac biomarker (lower panel) for detection of c) CK‐MB, d) Mb, and e) cTnI. Error bars indicate the standard deviation of the mean frequency shifts for the studied concentration.

Our studies went on to examine multiplexed detection of these three serum cardiac biomarkers on the plasmonic metasurface. While the quartz‐based plasmonic metasurfaces for separate cardiac biomarker detection have a dimension of 12 mm × 12 mm or larger, silicon substrates were used for the multiplexed detection studies, and these were diced into smaller substrate sections with a dimension of about 5 mm × 5 mm. The plasmonic metasurfaces were then fabricated onto these smaller silicon substrates following the fabrication protocol in Figure 1. Subsequently, they were functionalized with three types of Raman molecules and monoclonal antibodies, as schematically shown in **Figure** **5**a. In the meanwhile, 3D printing was utilized to create a compartmentalized biosensing platform (Figure 5b) with sectors measuring 6 mm × 6 mm, so that each compartment could hold a functionalized plasmonic metasurface substrate as shown in Figure 5a. The functionalized plasmonic metasurfaces were thereby integrated into the 3D printed biosensing platform, where each row had the same type of monoclonal antibody functionalization. Subsequently, serum samples containing a mixture of cardiac biomarkers including equal portions of CK‐MB, Mb, and cTnI were pipetted onto the multiplexed biosensing platform. After incubation at 37 °C for 20 min, excessive reagents were washed away using PBS buffer and then the sections were dried with compressed air. The integrated multiplexed biosensing platform was then characterized using Raman spectroscopy with a total of 5 × 5 spectra acquired over areas of 20 µm × 20 µm under an excitation wavelength of 785 nm.

As each row was functionalized with the same type of monoclonal antibodies, it was thus capable of capturing the corresponding cardiac biomarker in a highly specific manner despite the co‐existence of other two types of nonspecific biomarkers in the sample. The obtained SERS spectra from each row were averaged and plotted with a vertical offset in Figure 5c–e for detecting CK‐MB, Mb, and cTnI, respectively. Corresponding SERS spectra with an extended wavenumber range were presented in Figure S2 (Supporting Information). The visually discernible frequency shifts for the studied peak of interest with respect to varying concentrations of cardiac biomarkers confirmed the specificity for the multiplexed biosensing method. This is reinforced by the coefficient of determination R^2^ based on the regression analysis for the correlation between the frequency shift and the cardiac biomarker concentration, which are 0.97, 0.90, and 0.98 for detecting CK‐MB, Mb, and cTnI, respectively. Notably, the linear detection ranges from 0.3 to 33.3 ng/mL, 3.3 to 1333 ng/mL, and 10 to 9009 pg/mL obtained in the multiplexed manner also covered the cutoff values for these three serum cardiac biomarkers. Additionally, the LOD for multiplexed detection of CK‐MB, Mb, and cTnI was estimated to be about 0.05 ng/mL, 3.8 ng/mL, and 7.0 pg/mL, respectively.

We also performed specificity tests for detecting each of the targets by utilizing mixtures of serum samples containing several paired concentrations of the other two cardiac biomarkers introduced as interfering agents. For example, to test the specificity of detecting CK‐MB, we mixed in paired portions of serum samples containing Mb (concentrations are: 0, 50, 200, 800, and 2000 ng/mL) and cTnI (concentrations are: 0, 30.7, 144, 567, and 2293 pg/mL). The mixed serum samples were marked as M0–M4. M0 contained a mixture of paired portions of Mb and cTnI, both without any biomarkers. M1 contained a mixture of paired portions of Mb with a concentration of 50 ng/mL and cTnI with a concentration of 30.7 pg/mL. In the same manner, M4 contained a mixture of paired portions of Mb with a concentration of 2000 ng/mL and cTnI with a concentration of 2293 pg/mL. M0–M4 were then pipetted onto the row of the multiplexed biosensing platform that were functionalized with CK‐MB monoclonal antibodies (Figure 5b). Following the same incubation and detection protocol as presented in Figure 5b, the SERS spectra were obtained and averaged, which, along with the relative frequency shifts, were plotted in Figure S3a (Supporting Information). The relative frequency shift Δν was defined as Δν  = ν_0_  − ν_*i*_, where ν_*i*_ is the frequency measured for the sample *M_i_* (*i*  =  1,  2,  3,  4) and ν_0_ is that for the sample *M*_0_. Detailed protocols for the specificity tests for detecting Mb and cTnI are summarized in Section S4 (Supporting Information). Likewise, the SERS spectra and frequency shifts obtained in specificity tests for detecting Mb and cTnI are presented in Figure S3b,c (Supporting Information), respectively.

We observed that, for the specificity test of detecting CK‐MB (Figure S3a, Supporting Information), the mixture of Mb and cTnI biomarkers initially induced a slight frequency shift. The frequency shift plateaued with further interferent concentration increases. This suggested a limited interference from the mixture of Mb and cTnI biomarkers. For the specificity test for detecting Mb (Figure S3b, Supporting Information) and cTnI, while slight frequency shifts were initially induced, no further consistent frequency shifts were observed. By comparing Figure S3a–c (Supporting Information) with Figure 5c–e (Supporting Information), we can reasonably deduce that the observed frequency shifts in Figure 5c–e are more likely to be induced by the captured biomarkers through specific antibody‐antigen interaction rather than the interfering agents.

It is also important to note that the multiplexed biosensing method features a series of unique advantages. Specifically, it combines spatially extended and weakly wavelength‐dependent EM field profiles on the plasmonic metasurface for SERS enhancement while utilizing the single‐antibody strategy to capture cardiac biomarkers in a specific and cost‐effective manner. The nanomechanical perturbation‐induced frequency shift for signal readout is not subjected to the SERS intensity fluctuations, whereas the three different Raman molecules used can transduce spectrally differentiated SERS peaks for multiplexing without spectral interference. The 3D printing method used to create a compartmentalized biosensing platform significantly increases the detection efficiency on the same substrate. A comparison between our study and the prevailing SERS methods for detection of cardiac biomarkers, as summarized in Table S1 (Supporting Information), further highlights that our integrated SERS frequency‐shift method provides a comprehensive and innovative strategy based on SERS frequency shifts, rather than SERS intensity, for studying a panel of serum cardiac biomarkers with practically useful and clinically relevant performance.

## 3 Conclusion 

In this study, we first designed and then fabricated a hexagonally periodic plasmonic metasurface using nanosphere lithography. Each constituent element within the array was featured by a nanopyramidal meta‐atom consisting of alternately layered gold–silica thin films. FDTD numerical simulations revealed that the plasmonic metasurface supported spatially extended and overall weakly wavelength‐dependent SERS enhancement. Such superior SERS performance was utilized to first create a biosensing platform for separate detection of three types of serum cardiac biomarkers, including CK‐MB, Mb, and cTnI. Functionalized plasmonic metasurfaces were further integrated with a 3D printed biosensing platform for multiplexed detection of these three serum cardiac biomarkers in a specific manner from sample mixtures. Given the adaptability of this multiplexed biosensing platform, we envision that it can be extended as a general in vitro optical sensing platform for high‐throughput multiplexed analysis of a wide range of chemical and biological species, such as small molecules, proteins, peptides, and nucleic acids.

## Conflict of Interest

The authors declare no conflict of interest.

## Associated Content

Supporting Information: Sections S1–S6; Figures S1–S3; Table S1.

## Disclaimer

Commercial equipment and materials used/mentioned in this work are identified in order to adequately specify certain procedures. In no case does such identification imply recommendation or endorsement by the National Institute of Standards and Technology, nor does it imply that the materials or equipment identified are necessarily the best available for the purpose.

## Supporting information

Supporting Information

## Data Availability

The data that support the findings of this study are available from the corresponding author upon reasonable request.
