# Supplementary material for: Multiplexed SERS Detection of Serum Cardiac Markers Using Plasmonic Metasurfaces
Source: Adv Sci (Weinh). 2024 Oct 15;11(45):2405910. doi: 10.1002/advs.202405910 (PMC11615760; doi:10.1002/advs.202405910)
Supplement: Supplementary file 1 — Supporting Information [file ADVS-11-2405910-s001.docx]

Supporting Information

**Multiplexed SERS Detection of Serum Cardiac Markers Using Plasmonic Metasurfaces**

Peng Zheng^1,2^, Lintong Wu^1^, Piyush Raj^1^, Jeong Hee Kim^1^, Santosh Paidi^1^, Steve Semancik^2^, Ishan Barman^1,3,4*^

^1^Department of Mechanical Engineering, Johns Hopkins University, Baltimore, MD 21218, United States

^2^Biomolecular Measurement Division, Material Measurement Laboratory, National Institute of Standards and Technology, Gaithersburg, MD 20899, United States

^3^Department of Oncology, Johns Hopkins University School of Medicine, Baltimore, MD 21287, United States

^4^The Russell H. Morgan Department of Radiology and Radiological Science, Johns Hopkins University School of Medicine, Baltimore, MD 21287, United States

*To whom the correspondence should be addressed. E-mail: [ibarman@jhu.edu](mailto:ibarman@jhu.edu)

**Section S1 Chemicals**

4-Mercaptobenzoic acid (99%), 5,5’-Dithiobis (2-nitrobenzoic acid) (≥98%), and 6-Thioguanine (≥98%) were purchased from Sigma-Aldrich*. Creatine Kinase MB (CK-MB) Monoclonal Antibody and Myoglobin (Mb) Monoclonal Antibody were purchased from Scripps Laboratories*. Cardiac Troponin-I (cTnI) Monoclonal Antibody and PBS buffer solution (1×, pH 7.4, Catalog number: 10010023) were purchased from ThermoFisher Scientific*. CK-MB, Mb, and cTnI with varying concentrations in serum were provided by Beckman Coulter Inc*.

**Section S2 Fabrication of plasmonic metasurfaces**

As schematically laid out in Fig. 1a-d, fabrication of plasmonic metasurfaces started with cleaning the quartz substrate. This was done by immersing the quartz substrates into acid piranha under heating at 90 °C for 2 hours. Afterwards, the quartz substrates were rinsed in D.I. water and sonication in ethanol and D.I. water, respectively.

Prior to patterning of polystyrene (PS) beads onto the cleaned quartz slides, they were redispersed with a weight percentage of 10% in the mixture of water and ethanol with a volume ratio of 1:1. The PS beads (1 µm in diameter) were then transferred using dip coating to the cleaned quartz substrates and left to dry naturally. Afterwards, gold and silica thin layers (20 nm and 10 nm in thickness, respectively) were alternately deposited by e-beam evaporator to fill the gaps defined by the hexagonally patterned polystyrene beads. A total of five thin-film layers of gold and four layers of silica were alternately deposited. Because of the poor adhesion between the metal and dielectric, a thin layer of chromium with a nominal thickness of 5 nm was deposited as the initial step, and then an ultrathin layer of chromium with a nominal thickness of 2 nm was deposited between each subsequent gold and silica deposition. After removal of the PS beads by sonication in ethanol led to the formation of the pyramidal plasmonic metasurfaces.

**Section S3 Instrumentations and characterizations**

Mira 3 Tesscan* scanning electron microscopy (SEM) with an acceleration voltage of 10 kV was utilized to characterize the fabricated plasmonic metasurfaces. Raman spectroscopy characterizations were performed using an XploRA PLUS Raman microscope (HORIBA Instruments Inc.*, Edison, NJ, USA) with an excitation laser wavelength of 785 nm and an objective of 50×. The output power was measured to be about 0.4 µW.

**Section S4 3D printing**

A FDM* 3D printer (CR-10S Pro V2) was employed to perform 3D printing. Thermoplastic polyurethane (TPU) filament was used and heated to 228 ℃ at the extrusion nozzle for 3D printing. In Fig. 4b, TPU grids were printed on the surface of each of the three plasmonic metasurfaces. The printed compartments have a dimension of about 3 mm by 3 mm and are separated by a TPU wall with a thickness of about 1 mm or less. In Fig. 5b, a compartmentalized TPU-based platform was 3D-printed and consists of an array of sectors with each measuring 6 mm by 6 mm (Fig. 5b), so that each compartment could hold a functionalized plasmonic metasurface substrate as shown in Fig. 5a. The functionalized plasmonic metasurfaces were thereby integrated into the 3D printed TPU-based biosensing platform, where each row had the same type of monoclonal antibody functionalization.

**Section S5 Specificity test**

To perform specificity tests for detecting each of the targets by utilizing mixtures of serum samples containing several paired concentrations of the other two cardiac biomarkers introduced as interfering agents. For example, to test the specificity of detecting CK-MB, we mixed in paired portions of serum samples containing Mb (concentrations are: 0, 50, 200, 800, 2000 ng/mL) and cTnI (concentrations are: 0, 30.7, 144, 567, 2293 pg/mL). The mixed serum samples were marked as M0 to M4. M0 contained a mixture of paired portions of Mb and cTnI, both without any biomarkers. M1 contained a mixture of paired portions of Mb with a concentration of 50 ng/mL and cTnI with a concentration of 30.7 pg/mL. In the same manner, M4 contained a mixture of paired portions of Mb with a concentration of 2000 ng/mL and cTnI with a concentration of 2293 pg/mL. M0 to M4 were then pipetted onto the row of the multiplexed biosensing platform that were functionalized with CK-MB monoclonal antibodies (Fig. 5b). After incubation at 37 °C for 20 minutes, excessive reagents were washed away using PBS buffer and dried with compressed air. That particular row was then characterized using Raman spectroscopy with a total of 5 × 5 spectra acquired over areas of 20 µm × 20 µm under an excitation wavelength of 785 nm. The obtained averaged SERS spectra along with the relative frequency shifts are plotted in Fig. S3a. The relative frequency shift $\Delta\nu$ was defined as $\Delta\nu=\nu_{0}-\nu_{i}$, where $\nu_{i}$ is the frequency measured for the sample $M_{i} (i=1, 2, 3, 4)$ and $\nu_{0}$ is that for the sample $M_{0}$.

Likewise, to perform the specificity test of detecting Mb, we mixed in paired portions of serum samples containing CK-MB (concentrations are: 0, 3, 10, 30, 100 ng/mL) and cTnI (concentrations are: 0, 30.7, 144, 567, 2293 pg/mL). The mixed serum samples were also marked as M0 to M4. To perform the specificity test for detecting cTnI, we mixed in paired portions of serum samples containing CK-MB (concentrations are: 0, 3, 10, 30, 100 ng/mL) and Mb (0, 50, 200, 800, 2000 ng/mL). The mixed serum samples were similarly marked as M0 to M4. The obtained SERS spectra and relative frequency shifts are presented in Figs. S3b and S3c, respectively.

**Section S6 FDTD numerical simulations**

Ansys Lumerical FDTD* (release: 2021. R2; version: 8.26.2717) was utilized for numerical simulations. A total-field scattered-field (TFSF) was implemented as the input light source from 700 nm to 1000 nm. A mesh size of 1 nm was used. Perfectly matched layer boundary conditions were imposed in all directions. The background refractive index was set at 1.0, whereas the refractive index for quartz substrates was set at 1.45. The dielectric function for gold was extracted from Johnson and Christy.^1^

**NIST equipment/supplies disclaimer:**

* Commercial equipment and materials are identified in order to adequately specify certain procedures. In no case does such identification imply recommendation or endorsement by the National Institute of Standards and Technology, nor does it imply that the materials or equipment identified are necessarily the best available for the purpose.

**
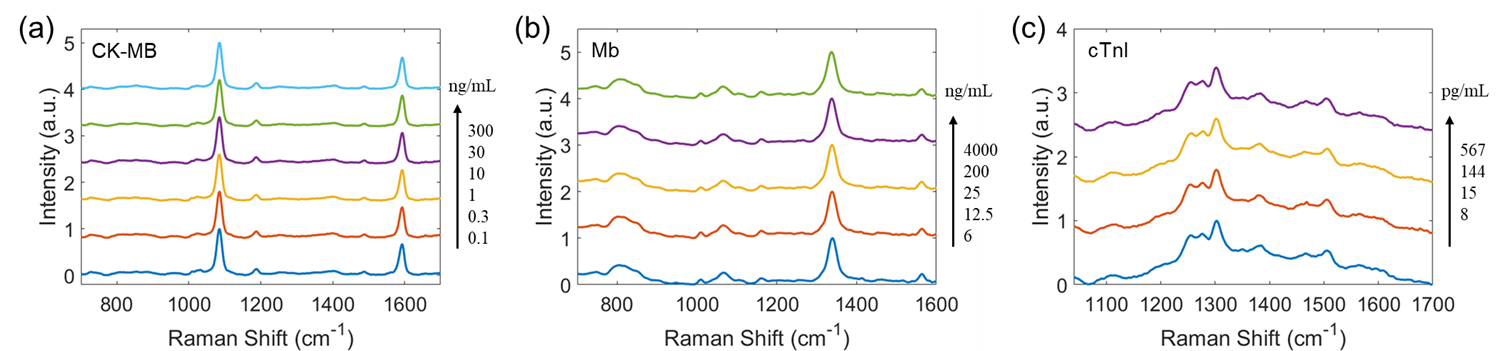
**

**Figure S1** Extended wavenumber range for SERS spectra collected for separate detection of each type of serum cardiac biomarkers. (a) SERS spectra of CK-MB monoclonal antibody-conjugated MBA after capturing CK-MB antigens with various concentrations, (b) SERS spectra of myoglobin monoclonal antibody-conjugated DTNB after capturing myogoblin antigens with various concentrations, and (c) SERS spectra of troponin-I monoclonal antibody-conjugated MP after capturing troponin-I antigens with various concentrations.

**
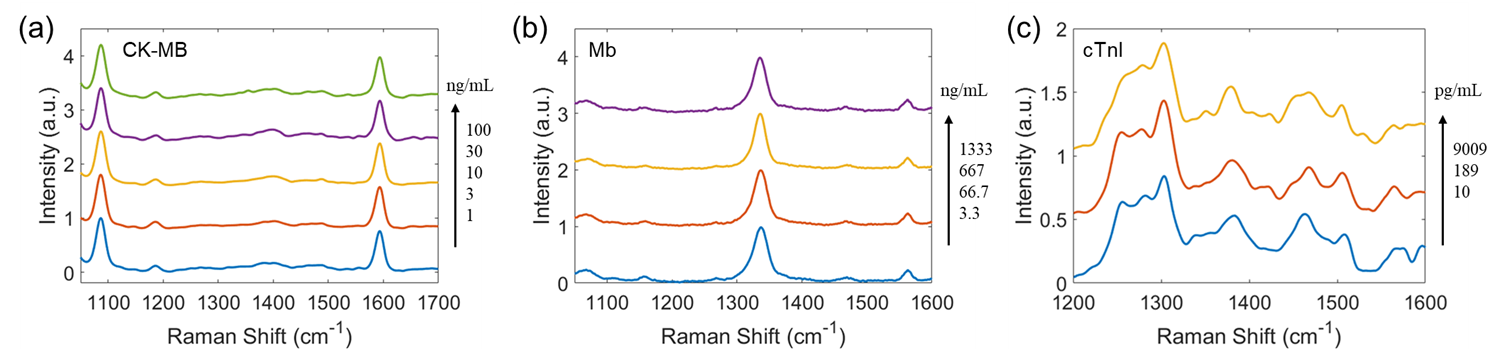
**

**Figure S2** Extended wavenumber range for SERS spectra collected for multiplexed detection of serum cardiac biomarkers. (a) SERS spectra of CK-MB monoclonal antibody-conjugated MBA after capturing CK-MB antigens with various concentrations, (b) SERS spectra of myoglobin monoclonal antibody-conjugated DTNB after capturing myogoblin antigens with various concentrations, and (c) SERS spectra of troponin-I monoclonal antibody-conjugated MP after capturing troponin-I antigens with various concentrations.

**
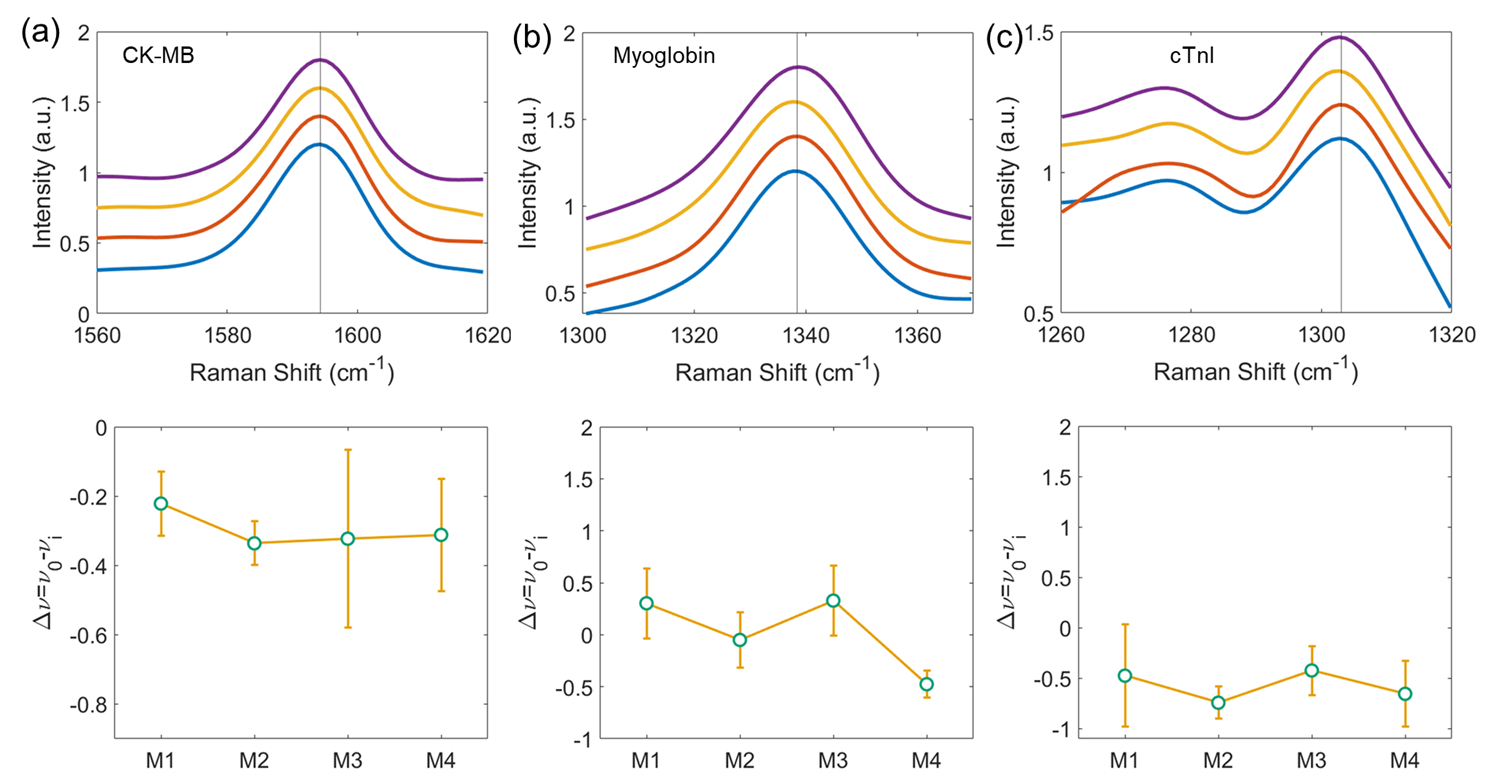
**

**Figure S3** Specificity test for detecting (a) CK-MB, (b) Mb, and (c) cTnI. The upper panel showed the averaged SERS spectra; the lower panel showed the corresponding frequency shifts. The relative frequency shift $\Delta\nu$ was defined as $\Delta\nu=\nu_{0}-\nu_{i}$, where $\nu_{i}$ is the frequency measured for the sample $M_{i} (i=1, 2, 3, 4)$ and $\nu_{0}$ is that for a sample $M_{0}$. The definition of M1 to M4 can be found in Section S4.

**Table S1** A comparison between our current study and prevailing SERS methods for detection of cardiac biomarkers. It is important to note that the normal concentrations for CK-MB, Mb, and cTnI biomarkers are 0.3 to 4 ng/mL^2^, 50 ng/mL or less^3^, and 40 pg/mL or less^2^. “NA” means being not applicable.

| Source | Method | Sample matrix | Separate detection range  (Limit of detection) | | | Multiplexed detection  (Limit of detection) | | |
| --- | --- | --- | --- | --- | --- | --- | --- | --- |
|  |  |  | CK-MB (ng/mL) | Mb (ng/mL) | cTnI (pg/mL) | CK-MB (ng/mL) | Mb (ng/mL) | cTnI (pg/mL) |
| Current study | SERS frequency shift | Serum | 0.1 - 300  (0.04) | 6 - 4000  (3.6) | 8 - 567  (5.2) | 0.3 - 33.3  (0.05) | 3.3 - 1333  (3.8) | 10 - 9009  (7.0) |
| Ref^4^ | SERS intensity | PBS buffer | 0.01 – 1000  (0.01) | NA | 0.01 – 1000  (0.01) | NA | NA | NA |
| Ref^5^ | SERS intensity | PBS buffer | 1 – 1000  (6.56 fg/mL) | NA | 100 - 1000000  (11.81 fg/mL) | 1 - 1000  (5) | NA | 100 – 1000000  (800) |
| Ref^6^ | SERS intensity | Serum | 0.01 – 50  (7.92 pg/mL) | NA | 10 - 50000  (2.94 pg/mL) | NA | NA | NA |
| Ref^7^ | SERS intensity | Serum | NA | NA | NA | 0.02 - 90  (0.7) | 0.01 - 500  (1) | 10 - 50000  (800) |
| Ref^8^ | SERS intensity | Urine | NA | 10 - 5000  (10) | NA | NA | NA | NA |
| Ref^9^ | SERS intensity | Serum | NA | 1 - 100000  (230) | NA | NA | NA | NA |
| Ref^10^ | SERS intensity | PBS buffer | NA | NA | 0 - 2000  (9.8) | NA | NA | NA |
| Ref^11^ | SERS intensity | Serum | NA | NA | 100 - 10000  (33.7) | NA | NA | NA |
| Ref^12^ | SERS intensity | Serum | NA | NA | 10 - 100000  (5.5) | NA | NA | NA |
| Ref^13^ | SERS intensity | Serum | NA | NA | 100 – 100000  (100) | NA | NA | NA |
| Ref^14^ | SERS intensity | Serum | NA | NA | 10 - 1000  (10) | NA | NA | NA |

**References**

1. Johnson, P. B.; Christy, R. W. *Physical Review B* **1972,** 6, (12), 4370-4379.

2. Park, J. H.; Heo, R.; Kang, H.; Oh, J.; Lim, T. H.; Ko, B. S. *Clin Exp Emerg Med* **2020,** 7, (3), 183-189.

3. de Winter, R. J.; Lijmer, J. G.; Koster, R. W.; Hoek, F. J.; Sanders, G. T. *Annals of Emergency Medicine* **2000,** 35, (2), 113-120.

4. Liu, Y.; Gao, R.; Zhuo, Y.; Wang, Y.; Jia, H.; Chen, X.; Lu, Y.; Zhang, D.; Yu, L. *Analytica Chimica Acta* **2023,** 1239, 340673.

5. Lee, H.; Kim, W.; Song, M.-Y.; Kim, D.-H.; Jung, H. S.; Kim, W.; Choi, S. *Small* **2024,** 20, (8), 2304999.

6. Gao, R.; Chen, F.; Yang, D.; Zheng, L.; Jing, T.; Jia, H.; Chen, X.; Lu, Y.; Xu, S.; Zhang, D.; Yu, L. *Sensors and Actuators B: Chemical* **2022,** 369, 132378.

7. Zhang, D.; Huang, L.; Liu, B.; Ni, H.; Sun, L.; Su, E.; Chen, H.; Gu, Z.; Zhao, X. *Biosensors and Bioelectronics* **2018,** 106, 204-211.

8. El-Said, W. A.; Fouad, D. M.; El-Safty, S. A. *Sensors and Actuators B: Chemical* **2016,** 228, 401-409.

9. Lin, C.; Li, L.; Feng, J.; Zhang, Y.; Guo, H.; Lin, X.; Li, R. *Analytica Chimica Acta* **2022,** 1225, 340253.

10. Hu, C.; Ma, L.; Mi, F.; Guan, M.; Guo, C.; Peng, F.; Sun, S.; Wang, X.; Liu, T.; Li, J. *New Journal of Chemistry* **2021,** 45, (6), 3088-3094.

11. Chon, H.; Lee, S.; Yoon, S.-Y.; Lee, E. K.; Chang, S.-I.; Choo, J. *Chemical Communications* **2014,** 50, (9), 1058-1060.

12. Wang, S.; Wang, C.; Xin, Y.; Li, Q.; Liu, W. *Microchimica Acta* **2022,** 189, (3), 125.

13. Khlebtsov, B. N.; Bratashov, D. N.; Byzova, N. A.; Dzantiev, B. B.; Khlebtsov, N. G. *Nano Research* **2019,** 12, (2), 413-420.

14. Lin, C.; Li, L.; He, Y.; Zhang, Y. *Chemistry Letters* **2022,** 51, (3), 303-307.
